# Generation of an *Lpar1*-EGFP Fusion Knock-in Transgenic Mouse Line

**DOI:** 10.1007/s12013-021-01033-5

**Published:** 2021-10-15

**Authors:** Richard Rivera, Nyssa A. Williams, Grace G. Kennedy, Paloma Sánchez-Pavón, Jerold Chun

**Affiliations:** 1 Translational Neuroscience Initiative, Sanford Burnham Prebys Medical Discovery Institute, La Jolla, CA, USA

## Abstract

Lysophosphatidic acid (LPA) is a lysophospholipid that acts as an extracellular signal through the activation of cognate G protein-coupled receptors (GPCRs). There are six known LPA receptors (LPA_1–6_). The first such receptor, LPA_1_, was identified in the embryonic brain and it has been studied extensively for gene expression throughout the body, including through studies of receptor-null knock-out mice. However, identifying receptor protein expression in situ and in vivo within living cells and tissues has been difficult because of biologically low receptor expression and variable antibody specificity. Towards visualizing native LPA_1_ receptors in situ, here we report the generation of a knock-in mouse produced by homologous recombination of murine embryonic stem (ES) cells to replace a wildtype *Lpar1* allele with a mutant allele created by in-frame fusion of EGFP to the 4th exon of *Lpar1* (*Lpar1-EGFP* knock-in allele). Homozygous knock-in mice appeared normal and at the expected mendelian ratios of knock-in allele transmission and females-to-males. Histological assessments of the fetal and adult central nervous system (CNS) demonstrated expression patterns that were consistent with prior in situ hybridization studies. This new mouse line will be useful for studies of LPA_1_ in the developing and adult CNS, as well as other tissues, and for receptor assessments in living tissues and disease models.

## Introduction

Molecular cloning of the first lysophospholipid receptor was reported 25 years ago from studies of the embryonic cerebral cortex [1], which identified a receptor now known as LPA_1_ (gene name *Lpar1* for mouse, *LPAR1* for human [2]) that mediates the effects of lysophosphatidic acid (LPA) [3–5]. In the ensuing years, other G protein-coupled receptors (GPCRs) for LPA and related lysophospholipids, including sphingosine 1-phosphate (S1P) were identified, revealing a rich biology with true medical relevance [5–7].

The identification of *Lpar1* from the central nervous system (CNS) implicated a range of potential functions relevant to normal and pathological states [3, 8–14]. During embryonic development, *Lpar1* was found to be expressed by neural progenitor cells (NPCs) of the ventricular zone, cells of the leptomeninges [1, 15] within the telencephalon, cells along the ventricular surface of the third ventricle [16], and at lower points in the neuraxis. Embryonic functions of *Lpar1* include effects on cellular morphology [17–20], process and growth cone outgrowth [4, 19–22], electrophysiological responses [23, 24], and neurogenesis through cell survival and anti-apoptotic effects [25–28]. In adult life, *Lpar1* shows prominent expression in non-neuronal cells of the brain, particularly oligodendrocytes and Schwann cells of the peripheral nervous system [26, 29–31]. Markedly lower levels of expression have been detected in most other brain cell types including microglia, astrocytes, endothelial, ependymal, and choroid cells, along with certain neurons, particularly under the varying conditions of development, disease, or cell culture [32–39].

Disruption of embryonic LPA_1_ signaling likely contributes to brain pathologies such as developmental brain disorders like hydrocephalus [16, 40] and neuropsychiatric disorders [41–45], including those associated with hypoxic mechanisms [46, 47]. During adulthood, other nervous system diseases have been linked to defects in LPA_1_ signaling including neuropathic and other forms of pain [42, 48–55], neuroinflammation [13, 56, 57], spinal cord injury [49, 52, 58, 59], stroke [60], and neurodegenerative disorders [13, 57].

Much of what is known about the physiology and pathophysiology of LPA_1_ came from experimental studies of the mouse, where the receptor was first cloned, through the targeted genetic deletion of *Lpar1* [27, 55], as well as by using the derivatives of the original mutant to improve viability in complex strain backgrounds (*maLpar1* [61]). A particular area of ambiguity has been ascertaining the biologically relevant locations of the LPA_1_ protein within the intact brain towards understanding cellular mechanisms. While gene expression studies by in situ hybridization (ISH) identified *Lpar1* expression in NPCs [1] during embryonic brain development, and oligodendrocytes [29] in the adult brain, determining the location of receptor protein has been more challenging because of inconsistent antibody specificity and availability, low receptor expression, differences in tissue preparation, and variables related to the effects of mouse background strain and husbandry that produced inconsistent, if not contradictory results. A possible solution to these challenges in mice is through gene knock-in strategies [62], wherein a wildtype allele is replaced by a modified version that enables tracking of a functional receptor tagged with an EGFP fusion protein. Here we report an *Lpar1-*EGFP fusion knock-in transgenic mouse line and its initial characterization within the developing and adult brain.

## Materials and Methods

### Mice

Animal husbandry for the *Lpar1*-*EGFP* mouse line was provided by the animal resource departments of The Scripps Research Institute (TSRI) and the Sanford Burnham Prebys Medical Discovery Institute (SBP). Embryonic and adult mice were euthanized via isoflurane overdose per approved protocols and institutional guidelines. All animal procedures were approved and conducted in accordance with the Institutional Care and Use Committee (**ICAUC**) guidelines of TSRI and SBP.

### ES Cell Transfection

Standard methodologies were used to establish *Lpar1-EGFP* knock-in ES cell lines [21, 27, 51, 55, 63–65]. Briefly, the targeting vector (50 mg) was linearized and mixed with 1 × 107 R1 ES cells (generously provided by Dr. Andras Nagy) in a 0.4 cm electroporation cuvette and electroporated using the Bio-Rad Gene Pulser II (200 mVolts × 800 mF capacitance). The electroporated cells were allowed to recover on ice for 20 min and then plated on a feeder layer of neomycin-resistant mouse embryo fibroblasts. Twenty-four hours after plating, the ES cell media was replaced with media containing 150 mg/ml of geneticin (ThermoFisher Scientific), which was replaced daily for 7 days. After 7 days, 140 individual ES cell clones were picked and split into 96 well master plates that were frozen in fetal calf serum containing 10% DMSO and 24 well plates for DNA isolation. Appropriately recombined ES clones were isolated and injected into recipient blastocysts by the TSRI Murine Genetics Core. Southern blotting and PCR genotyping with targeted primers identified germline transmission of the knock-in transgene (Fig. 2B: A1 EGFP KI Forward: 5′-GACAAAGAGATGAGCGCCAC-3′, A1 EGFP Wt Reverse: 5′-GAGTGTCCTCATCTCCTCTG-3′, and EGFP Internal Reverse: 5′-GTGGTGCAGATGAACTTCAGG-3′).

### Southern Blotting and DNA Hybridization

ES cells with homologous recombination of the *Lpar1* genomic locus were identified by Southern blot hybridization [51, 55, 66, 67] of *HindIII* digested genomic DNA that was separated on a 0.08% 1 × TAE agarose gel and transferred to Hybond^™^-N membranes (Amersham), UV-crosslinked, and probed with 25 ng of a ^32^P-labeled 1.2 kb DNA fragment from a region of the *Lpar1* locus external to 3′ end of the *Lpar1*-EGFP targeting construct. Blots were pre-hybridized for one hour at 42 °C in hybridization solution (0.5 M phosphate buffer pH 7.4, 50% formamide, 5 × SSPE, 1 × Denhardt’s solution, 1% SDS, and 0.1% denatured salmon sperm) followed by the addition of the denatured radiolabeled DNA probe prepared using an Agilent Random Primer Labeling Kit. Following overnight hybridization (42 °C), the blots were washed with 2 × SSPE 0.1% SDS and 0.2 × SSPE 0.1% SDS, then visualized with a GE Typhoon Phosphorimager. ES cell clones that did not have proper recombination of the *Lpar1* locus were identified by a 6.7 kb band, and ES cell clones with homologous recombination events at *Lpar1* were identified by the presence of a 6.7 kb wildtype band and 4.4 kb recombined band (Fig. 2A).

### Tissue Preparation and Sectioning

Mutant mice were identified and validated by standard genotyping procedures. Briefly, DNA was isolated from the tails of the offspring and two primer pairs were used to PCR screen for the presence (EGFP Forward 5′-ACATGGTCCTGCTGGAGTTC-3′ and Neo Reverse 5′-TGGATGTGGAATGTGTGCGAG-3′) and absence (EGFP Forward plus *Lpar1* Reverse 5′-GAGTGTCCTCATCTCCTCTG-3′) of the neomycin cassette. All tissue samples from employed animals showed EGFP fluorescence. Homozygote and heterozygote cryostat sections were then analyzed by histology. Briefly, expression of the *Lpar1-EGFP* fusion allele was assessed by isolating embryonic day 13.5 (E13.5) heads and adult brain and lumbar spinal cord from wildtype and *Lpar1-EGFP* knock-in age-matched animals, which were processed for standard cryostat sectioning, fixation, and fluorescence microscopy [68–73]. Samples were mounted in pre-chilled molds containing pre-chilled Neg-50^™^ (Thermo Scientific) frozen section media and then frozen on dry ice. Tissue sections were cut at 20 μm using a Leica cryostat, fixed with cold 4% paraformaldehyde in phosphate-buffered saline (PBS) for 5 min, washed twice with PBS, mounted, and coverslipped using VECTA-SHIELD^®^ HardSet^™^ antifade mounting medium with DAPI (Vector Laboratories, Burlingame, CA). Tissue section, staining, and mounting was performed at the SBP histology core facility. Endogenous EGFP fluorescence in all tissues was evaluated using a KEYENCE BZ-X810 all-in-one fluorescence microscope. RNAscope [74] against mouse *Lpar1* utilized commercially available probes and protocols (Advanced Cell Diagnostics, Newark, CA).

## Results

### Construction of an Lpar1-EGFP Fusion Knock-in Construct

Mouse *Lpar1* genomic DNA fragments were amplified from a bacterial artificial chromosome (BAC) template containing the *Lpar1* genomic locus (BAC RP23–149020, Children’s Hospital Oakland Research Institute) using Pfx50^™^ DNA polymerase (Invitrogen). An amplified fragment containing *Lpar1* exon 4 including a portion of the 3′ untranslated region (UTR) was used to fuse EGFP, in-frame, to the carboxy terminus of *Lpar1* using overlap PCR. An introduced *BamHI* restriction enzyme site positioned between the EGFP stop codon and the start of the *Lpar1* 3′ UTR was used to insert a loxP flanked neomycin cassette under the control of the phosphoglycerate kinase promoter (PGK-neo). A *HindIII* restriction enzyme at the 5′ end of the PGK-neo cassette was inserted to allow for the identification of homologously recombined ES cell clones. Amplified *Lpar1* genomic fragments were assembled in the pGEM^®^-T Easy Vector System (Promega) to produce an *Lpar1* knock-in targeting vector with 8.2 and 1.7 kb of homologous flanking sequence on the 5′ and 3′ ends of the EGFP and PGK-neo DNA sequences.

### Isolation of Recombined ES Cell Clones and Lpar1-EGFP Knock-in Mice

**Fig. 1:**
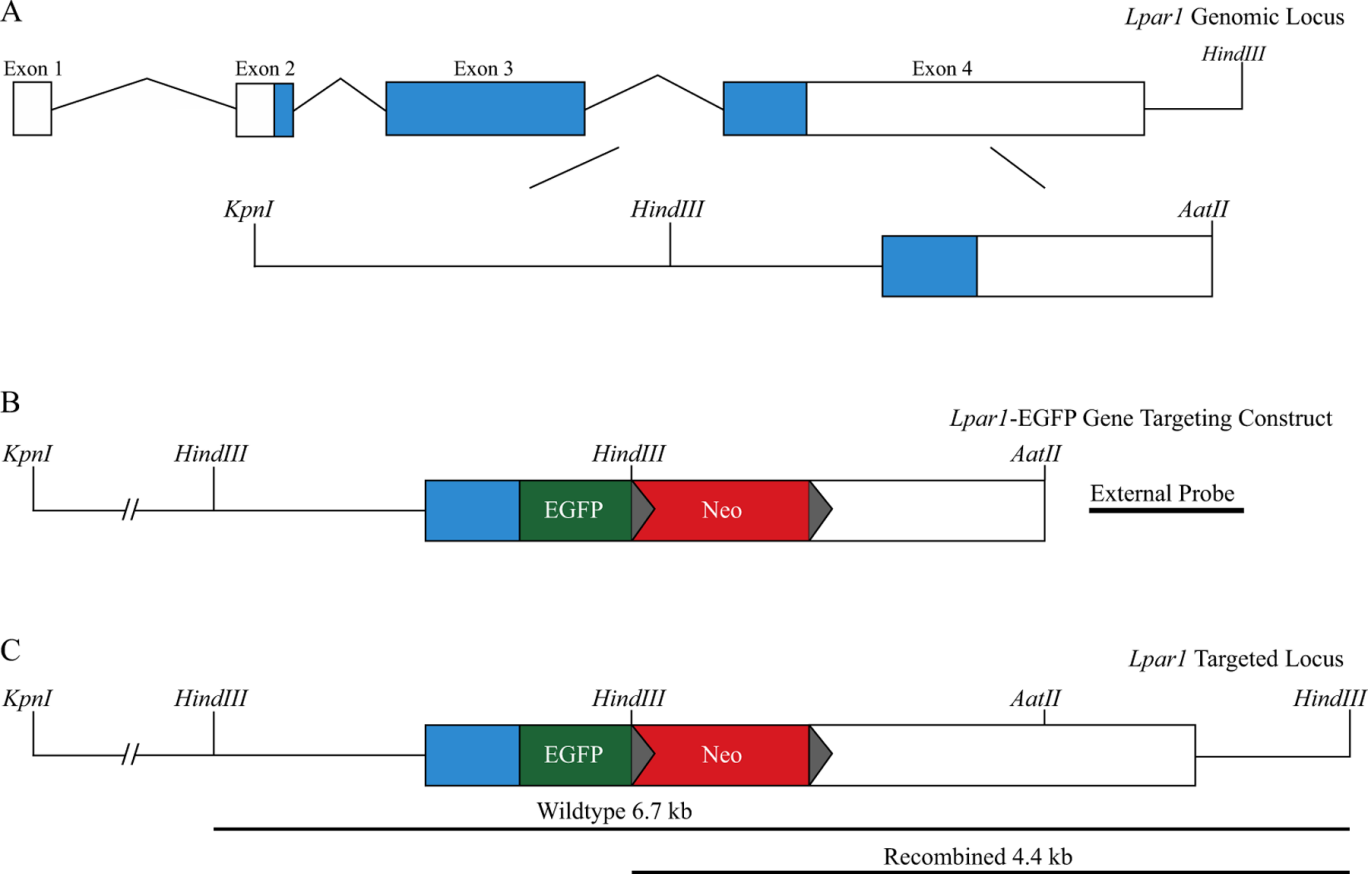
Schematic for the creation of the *Lpar1*-EGFP knock-in mouse by homologous recombination. **A** The *Lpar1* genomic locus and an expanded view of the region selected for gene targeting. The targeted region is a ~10 kb DNA fragment and it is flanked by a *KpnI* restriction enzyme site on the 5′ end and a 3′ *AatII* restriction enzyme site present in exon 4. **B** The *Lpar1*-EGFP knock-in targeting construct. EGFP is fused in-frame to the 3′ end of *Lpar1* (green) and the fusion removes the *Lpar1* stop codon and the EGFP start codon. A neomycin cassette (red) flanked by loxP sites (gray triangles) is inserted 3′ of the *Lpar1*-EGFP fusion. A *HindIII* restriction enzyme site is included in the neomycin cassette to distinguish non-recombined (wildtype) *Lpar1* alleles versus *Lpar1* alleles that have been replaced through homologous recombination. The external probe used for identifying homologous verses non-homologous recombination is 3′ to the *AatII* restriction enzyme site in the *Lpar1* locus. **C** The *Lpar1* targeted locus shows the predicted DNA size fragments of wildtype and homologously recombined *Lpar1* alleles. Southern blot hybridization of genomic DNA digested with *HindIII* and probed with the indicated external probe (**B**), will produce bands of 6.7 kb for non-homologously targeted alleles, and 4.4 kb for alleles that have undergone successful homologous recombination. The shorter 4.4 kb fragment observed in *Lpar1* alleles with homologous recombination is due to an introduced *HindIII* restriction enzyme site in the neomycin cassette.

**Fig. 2:**
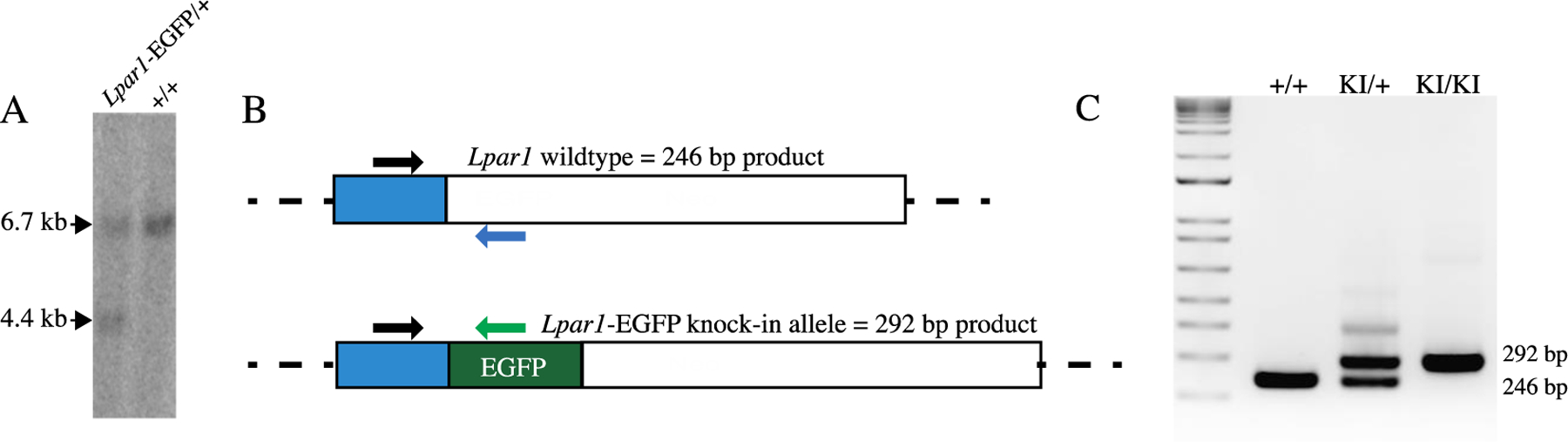
Confirmation of targeted ES cells and mice. **A** Southern blot of genomic DNA from ES cells electroporated with the targeting construct, digested with *HindIII*, and probed with the external probe. An ES cell clone with a successful homologous recombination event (*Lpar1-EGFP*/ +), indicated by hybridizing bands of 6.7 kb (wildtype) and 4.4 kb (homologously recombined), and an ES cell clone with a random integration event, indicated by the presence of a 6.7 kb wildtype hybridizing band only, are shown. **B** Schematic of the PCR genotyping primers for *Lpar1-EGFP* knock-in and wildtype mice. The three primer PCR genotyping reaction will produce bands of 246 bp for wildtype mice (blue reverse primer) and 292 bp for mice with a *Lpar1-EGFP* knock-in allele (green reverse primer). **C** 1.8% agarose gel showing PCR products for mice genotyped with the primers defined in **B**: wildtype (+/+), heterozygous knock-in (KI/ +), and homozygous knock-in mice (KI/KI).

ES cell clones positive for homologous recombination were identified by Southern blotting using the external probe (Fig. 2A). Positive clones were expanded for confirmation of the recombination event, analyzed to ensure that only single integration events were present, and pathogen tested (IDEXX BioAnalytics). A single ES cell clone was selected for injection into C57BL/6 J mouse blastocysts to produce chimeric mice. Chimeric offspring were bred to C57BL/6 J animals to assay for germline transmission. Homologous recombination events were assessed by Southern blot hybridization of *HindIII* digested genomic DNA isolated from the offspring of chimeric mice using the external probe (Fig. 1) and by PCR genotyping.

To delete the loxP flanked PGK-neo cassette, male mice heterozygous for the *Lpar1-EGFP* allele were crossed to female EIIa-cre transgenic mice that are routinely used for germline deletion of loxP-flanked DNA sequences [75–78]. Mice hemizygous for the *Lpar1-EGFP* allele and devoid of the neomycin cassette were identified by genomic Southern blotting (Fig. 2A) and PCR (Fig. 2B, C). Mice with appropriate genotypes were used to propagate the *Lpar1-EGFP* line and were bred to homozygosity. Homozygous animals appeared phenotypically indistinguishable from littermate controls, showed normal Mendelian female-to-male ratios, and were themselves fertile, allowing colony establishment with homozygous *Lpar1-EGFP* genotype-confirmed animals. Timed-breedings to assess embryonic day 13.5 (E13.5) brains, an age at which the ventricular zone of the embryonic cerebral cortex is prominent [1, 79, 80], were complemented by live births to produce adults for analyses.

### Histological Analyses Reveal a Concordance between Lpar1-EGFP and RNAscope

**Fig. 3:**
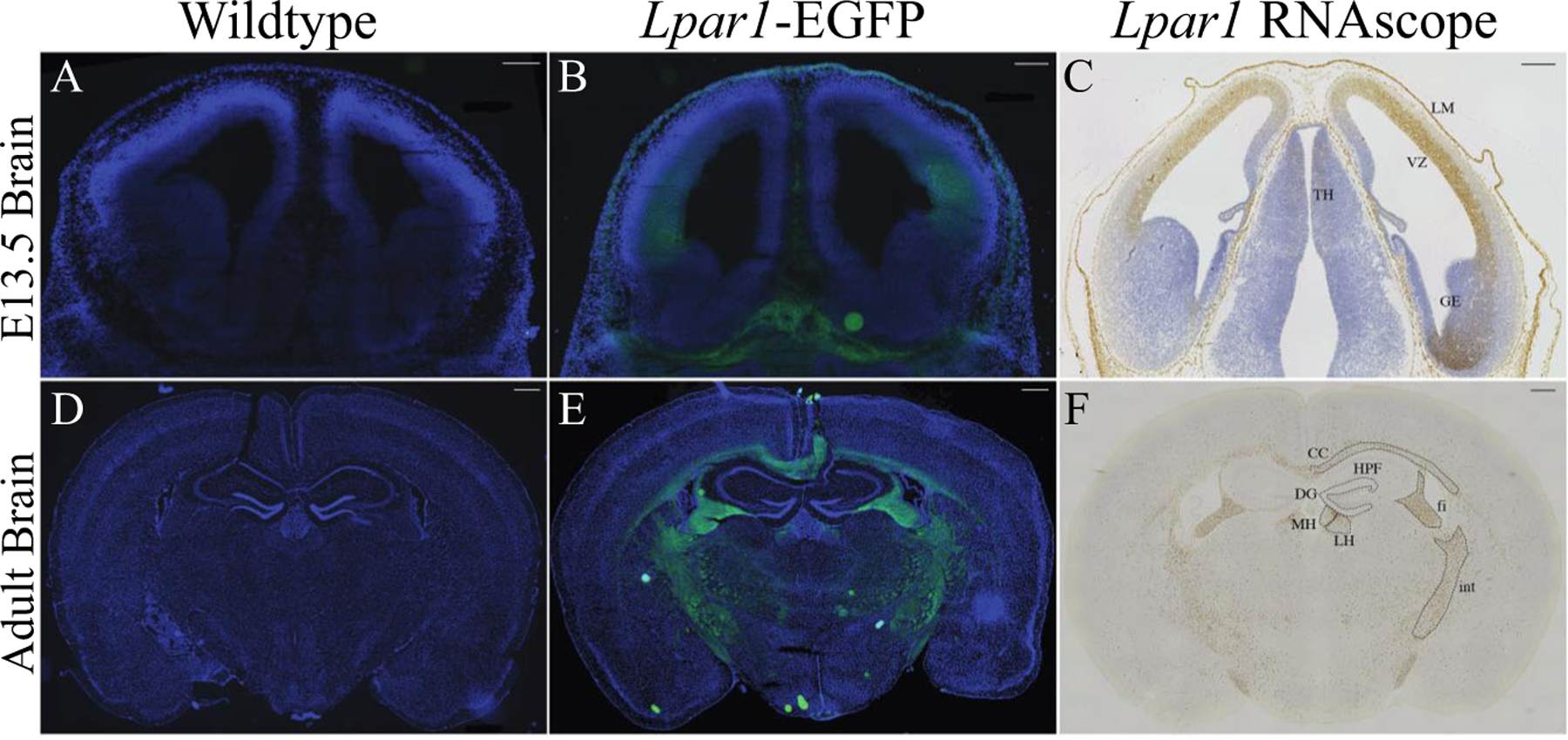
Endogenous EGFP fluorescence in tissues from adult and embryonic mice with *Lpar1-EGFP* alleles correlates with *Lpar1* RNA expression. **A**, **B** Coronal head sections from E13.5 wildtype and *Lpar1*^*EGFP/EGFP*^ mice showing LPA_1_ expression (green). Endogenous EGFP expression in *Lpar1*-EGFP knock-in mice (**A**, **B**) closely parallels (**C**) *Lpar1* mRNA expression in wildtype E13.5 mice that show *Lpar1* expression in the thalamus (TH), leptomeninges (LM), ganglionic eminence (GE), and the ventricular zone (VZ). **D**, **E** Coronal brain sections from 8-week-old wildtype and *Lpar1*^*EGFP/*+^ mice. EGFP fluorescence in the adult brain was primarily subcortical and correlates with (**F**) *Lpar1* mRNA expression in white matter areas including the corpus collosum (CC), internal capsule (int), fimbria (fi), medial habenula (MH), and lateral habenula (LH). Notably, *Lpar1* expression is not detected in the hippocampal formation (HPF) or dentate gyrus (DG). Scale bars = 200 μM for A, B, C and 500 μM for D, E, F. All images are shown at 10X magnification.

**Fig. 4:**
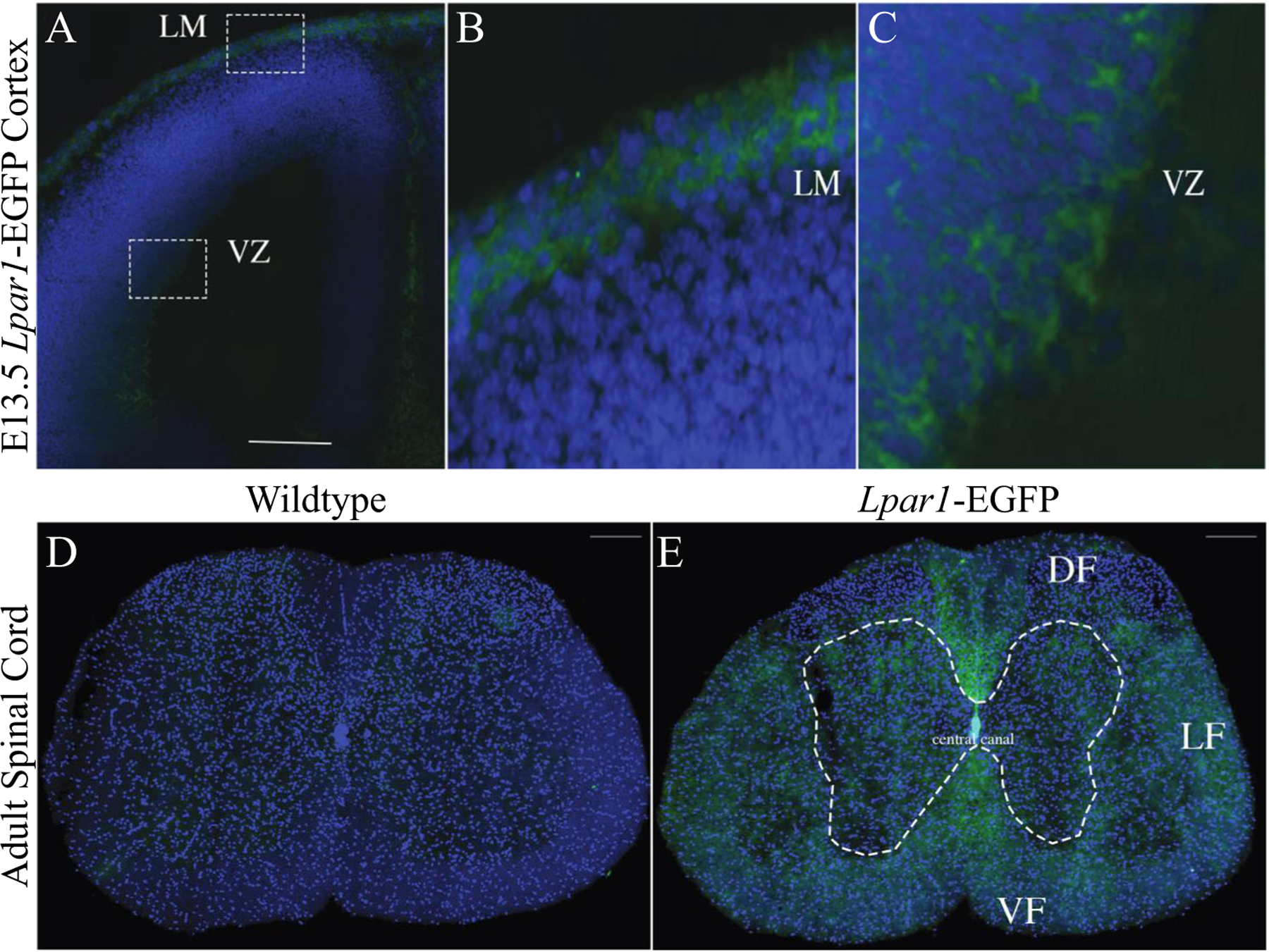
*Lpar1*-EGFP expression occurs on the cell surface. **A** The lateral ventricle of an E13.5 *Lpar1*^*EGFP/EGFP*^ mouse embryo, boxed areas highlight EGFP fluorescence in the leptomeninges (LM) and the ventricular zone (VZ). **B**, **C** Enlarged areas from the boxed regions in (**A**) of the LM and VZ show that *Lpar1*-EGFP is expressed on the cell surface. **D**, **E** Sections of lumbar spinal cord from 10-week-old wildtype and *Lpar1*^*EGFP/*+^ mice. EGFP expression in (**E**) shows LPA_1_ is highly expressed in white matter rich areas of the adult spinal cord including the dorsal funiculus (DF), lateral funiculus (LF), and dorsal funiculus (DF). The spinal cord central canal is shown for reference. Images shown are at 40X magnification for the E13.5 mouse cortex and 10X magnification for the spinal cord, scale bars = 200 mM.

Wildtype controls were compared to *Lpar1-EGFP* animals, wherein cryostat sections were mounted, fixed, counter-stained with 4,6-diamidino 2-phenylindole (DAPI) and observed using fluorescence microscopy (Fig. 3A, B). Specific EGFP endogenous fluorescence was observed only within mutants. In embryonic specimens, EGFP was detected in the cerebral cortical ventricular zone (VZ), leptomeninges (LH), and in non-brain compartments. Compared to *Lpar1* RNAscope in situ hybridization (Fig. 3C), the patterns of EGFP labeling extensively overlapped, including a relative absence of signal in the ganglionic eminence (ge), as previously observed [1, 16]. These results support the presence of higher receptor density on the cell membranes of soma expressing LPA_1_. Within the adult brain, strong EGFP labeling was observed within most, if not all, myelinated fiber tracts and prominently within the corpus callosum (CC), internal capsule (ic) fimbria (fi), and medial and lateral habenulae (MH, LH) (Fig. 3D–F). Notably, robust EGFP was not observed in the hippocampal formation (H) that included the dentate gyrus (dg). Further analyses supported EGFP localization surrounding cell nuclei (Fig. 4A–C), which is consistent with the membrane localization of LPA_1_. The spinal cord (lumbar region) was also examined (Fig. 4D, E), which revealed more diffuse EGFP signals within known myelinated fiber tracts.

## Discussion

Knowledge of LPA_1_ protein localization under basal conditions of normal development and adult life will enable more accurate mechanisms to be identified for biological, pathophysiological, and drug discovery efforts that target LPA_1_. The *Lpar1-EGFP* knock-in mice described here express from the endogenous promoter and enhancer elements, and show no evidence of LPA_1_ functional loss or deficiency compared to phenotypes from constitutive [21, 27, 61] and conditional [55] *Lpar1* knock-out studies. These results support the overall functionality of LPA_1_-EGFP fusion proteins which is consistent with other functional LPA_1_ fusion proteins, including their use to determine the LPA_1_ crystal structure (using the modified apocytochrome c, “bRil”) [81]. The presence of EGFP in non-brain compartments (Fig. 3B) indicates that this mutant will likely be useful for many, if not all, cell types that express LPA_1_. Formal examination of the other cell types and tissues known to express LPA_1_ [12] remains an area for future work.

The high concordance between EGFP signals and ISH labeling supports proximity between *Lpar1* mRNA and receptor protein, at least at the level of signal that can be identified by the employed techniques. This was particularly true in the embryonic brain where immature cells have relatively little cytoplasm compared to their nuclei [82], revealing EGFP signals surrounding nuclei (Fig. 4B, C). However, it is certain that minute levels of receptor protein expression, not reported by direct *Lpar1-EGFP* expression, are present, particularly in developing neurons and comparatively rare but existent neuronal expression of LPA_1_ that has been detected by single-cell Drop-seq transcriptomic studies [39]. Functional support for active receptors, at distal cellular locations of processes and growth cones on developing NPCs and neurons, can be seen in the effects of exogenous LPA exposure that results in process and growth cone retraction [18, 20, 22, 25, 83, 84], indicating that receptors are present in these highly polarized cells. Amplification techniques such as anti-EGFP antibodies and structured illumination microscopy [85] could better resolve the presence of low-abundance receptor signals marked by the expressed receptor fusion proteins. In the adult brain, oligodendrocytes were by far the most prominent cell type identified by early ISH studies [29]. This observation was confirmed by single-cell Drop-seq studies where the top 80+ subclusters with the highest *Lpar1* expression were predominantly oligodendrocytes (and other non-neuronal cell types) [39]. The robust labeling of myelinated fiber tracts is consistent with prior ISH and single-cell transcriptome studies that identified receptor expression in oligodendrocytes, while the current images support high-density LPA_1_ localization within the cell membrane layers of the myelin sheaths [26, 29, 86, 87].

An area of technical and biological ambiguity that may be approached using the newly created *Lpar1-EGFP* knock-in mice relates to the hippocampus and LPA_1_’s roles in learning and memory. Multiple reports identified hippocampal phenotypes in mice null for *Lpar1* [45, 88–91]. However, the cell types responsible for these phenotypes are ambiguous. One attractive cell population for future studies is the mouse adult hippocampal precursor cell [92–94] that was reported to show high levels of LPA_1_ expression based primarily upon the EGFP BAC mouse line created by the NIH GENSAT [95, 96] project. This mouse showed high EGFP expression within white matter tracts like the *Lpa*_*1*_-*EGFP* mice reported here, but also showed high EGFP expression within the dentate gyrus [94] (and other locales such as the cerebral cortex) that did not overlap with the *Lpa*_*1*_-*EGFP* expression pattern. This raises the question on which mouse model provides a more accurate description of LPA_1_ location.

Multiple, independent ISH analyses have uniformly been devoid of robust *Lpar1* signals in the dentate gyrus including the original GENSAT ISH screen, the Allen Brain Atlas, the current report (Fig. 3), and past reports [29], as well as Drop-seq data [39] that did report a dentate gyrus neuron subcluster (Neuron_dentate_C1qI2[#4](HC)), but with low expression levels (~2 *Lpar1* transcripts per million (TPM)) compared with the top brain subcluster (oligodendrocyte_Mbp[#9](TH); ~1600 *Lpar1* TPM). A putative hippocampal progenitor subcluster that is not limited to the dentate gyrus (Neurogenesis_Sox4 [#13](HC)) reported somewhat higher expression (~11 *Lpar1* TPM). Primary cell culture studies also reported an absence of LPA_1_ expression in hippocampal neurons [84].

Evidence for low LPA_1_ expression in the dentate gyrus vs. high EGFP expression in the BAC mice—as well as other discordant EGFP patterns—could be explained in multiple ways. A partial list includes the precise ages of brain analyses, tissue preparation, different EGFP half-life, differing mouse background strains, and mouse husbandry conditions, as well as a BAC-transgene that has unknown copy number, orientation, intactness, and integration sites. These issues might affect BAC-transgene EGFP expression under basal and challenged (e.g., cell culture, disease models) conditions. As notable, BAC-mouse lines were purposefully selected for optimal EGFP expression during line curation, which therefore selected for discrepant patterns compared to standard approaches [96] and might have for-gone lesser signals more akin to those seen in the current *Lpar1-EGFP* mouse that employs wildtype gene regulatory elements. Use of these new *Lpar1-EGFP* knock-in mice should enable future clarification of hippocampal phenotypes and related issues, while providing a new experimental tool to assess LPA_1_ protein localization within intact tissues.
